# Characterization of Shear-Sensitive Genes in the Normal Rat Aorta Identifies Hand2 as a Major Flow-Responsive Transcription Factor

**DOI:** 10.1371/journal.pone.0052227

**Published:** 2012-12-20

**Authors:** Hanna M. Björck, Johan Renner, Shohreh Maleki, Siv F. E. Nilsson, Johan Kihlberg, Lasse Folkersen, Matts Karlsson, Tino Ebbers, Per Eriksson, Toste Länne

**Affiliations:** 1 Division of Cardiovascular Medicine, Department of Medical and Health Sciences, Faculty of Health Sciences, Linköping University, Linköping, Sweden; 2 Center for Medical Image Science and Visualization (CMIV), Linköping University, Linköping, Sweden; 3 Division of Applied Thermodynamics and Fluid Mechanics, Department of Management and Engineering, Linköping University, Linköping, Sweden; 4 Atherosclerosis Research Unit, Center for Molecular Medicine, Department of Medicine, Karolinska Institute, Solna, Sweden; 5 Division of Drug Research, Department of Medical and Health Sciences, Faculty of Health Sciences, Linköping University, Linköping, Sweden; 6 Division of Radiology, University Hospital in Linköping, Linköping, Sweden; 7 University Hospital in Linköping, Linköping, Sweden; University of Otago, New Zealand

## Abstract

**Objective:**

Shear forces play a key role in the maintenance of vessel wall integrity. Current understanding regarding shear-dependent gene expression is mainly based on *in vitro* or *in vivo* observations with experimentally deranged shear, hence reflecting acute molecular events in relation to flow. Our objective was to combine computational fluid dynamic (CFD) simulations with global microarray analysis to study flow-dependent vessel wall biology in the aortic wall under physiological conditions.

**Methods and Results:**

Male Wistar rats were used. Animal-specific wall shear stress (WSS) magnitude and vector direction were estimated using CFD based on aortic geometry and flow information acquired by magnetic resonance imaging. Two distinct flow pattern regions were identified in the normal rat aortic arch; the distal part of the lesser curvature being exposed to low WSS and a non-uniform vector direction, and a region along the greater curvature being subjected to markedly higher levels of WSS and a uniform vector direction. Microarray analysis identified numerous novel mechanosensitive genes, including *Trpc4* and *Fgf12*, and confirmed well-known ones, e.g. K*lf2* and *Nrf2*. Gene ontology analysis revealed an over-representation of genes involved in transcriptional regulation. The most differentially expressed gene, *Hand2*, is a transcription factor previously shown to be involved in extracellular matrix remodeling. HAND2 protein was endothelial specific and showed higher expression in the regions exposed to low WSS with disturbed flow.

**Conclusions:**

Microarray analysis validated the CFD-defined WSS regions in the rat aortic arch, and identified numerous novel shear-sensitive genes. Defining the functional importance of these genes in relation to atherosusceptibility may provide important insight into the understanding of vascular pathology.

## Introduction

Biomechanical forces, generated by pulsatile blood flow, play a key role in the maintenance of vessel wall integrity as well as in the pathogenesis of vascular disease. Uniform flow generates high magnitudes of wall shear stress (WSS) and induces a distinct anti-proliferative and anti-inflammatory endothelial phenotype, along with induction of atheroprotective genes, such as *eNOS* and *Klf2* [1]. Disturbed flow with associated low magnitudes of WSS, on the other hand, renders the endothelium to become dysfunctional by increasing the expression of pro-inflammatory mediators, such as *NF-κB*, *TNF* and *VCAM1,* as well as genes related to pro-oxidation and pro-proliferation [2].

The impact of different patterns of WSS on vascular biology has been extensively studied *in vitro* or *in vivo* with experimental deranged shear [3], [4], [5], [6], [7]. Although *in vitro* studies have provided us with considerable useful information regarding shear-dependent mechanisms, experimental cell culture systems are highly simplified and may not fully represent genes/pathways involved *in vivo*. Indeed, in a study by Ni and colleagues, only about 50% of the mechanosensitive genes found *in vivo* could be replicated *in vitro* [8], demonstrating the critical need of *in vivo* models when studying shear-dependent vascular biology. *In vivo* models with experimentally deranged shear are limited to evaluation of acute molecular events associated with flow disturbances, and does not allow for the capture of compensatory mechanisms operating over longer exposure times, which is more relevant for vascular disease progression. A few studies addressing the “chronic” effect of shear stress by the use of image based computational fluid dynamics (CFD) to identify anatomically separated regions being exposed to different flow patterns have been performed [9], [10], but these studies have mainly focused on endothelial biology. Although endothelial cells (ECs) are the main sensors of fluid flow, they are in a complex crosstalk with the underlying tissue [11], [12], [13], in which upcoming pathological changes become mostly apparent, inducing phenotypic changes of vascular smooth muscle cells, enabling migration and extracellular matrix (ECM) remodeling.

In the present study, we combined CFD simulations of WSS with global gene expression analysis with the aim to investigate local shear-dependent vessel wall biology in the aortic arch of rat under physiological conditions. Aortic geometry and ascending aortic flow information were acquired using magnetic resonance imaging (MRI), followed by CFD estimation of WSS magnitude and WSS vector direction. Microarray analysis was carried out using RNA obtained from portions of the entire aortic wall (including intima, media end adventitia) exposed to high and low WSS, respectively. Gene ontology analysis was further performed demonstrating an over-representation of genes involved in transcriptional regulation. In particular, the basic helix-loop-helix (bHLH) transcription factor *Hand2* was markedly up-regulated in the endothelium of regions exposed to low WSS with disturbed flow, as shown by immunostaining.

## Materials and Methods

### Animals and Ethics Statement

Male Wistar rats (Taconic, Lille Skensved, Denmark) weighing 400–450 g were used. The experimental protocol was approved by the Regional Ethics Committee for Animal Experiments at Linköping University, Sweden, (Permit Number: 49-07) and strictly followed recommended guidelines for care and treatment of experimental animals. Animals were maintained at Animal Facility, Faculty of Health Sciences, Linköping University under constant environmental temperature (21°C) and 12 h:12 h light/dark cycle, and had free access to standard rodent chow and water.

### Experimental Procedure

WSS magnitude and WSS vector direction was determined in totally nine rats. The rats were anesthetized by intraperitoneal injection of a mixture of Rompun (10 mg/kg) and Ketalar (100 mg/kg). A femoral vein was then cannulated for intravenous administration of fluids. Additional doses of anesthetics were given subcutaneously or intravenously at regular intervals. Animals were then transported to the Centre for Medical Imaging and Visualization for determination of aortic geometry and three-dimensional blood flow. Rats were placed prone in the MRI scanner and a heat pack was used to maintain body temperature. Needle electrodes were inserted subcutaneously on all four limbs for registration of ECG, and an air filled cuff was placed under the chest of the animal to monitor respiration (MRI-compatible physiological monitoring and gating system for small animals, SA Instruments, Inc., Stony Brook, NY). A camera was used for continuous observation. Blood was obtained by heart puncture in a second set of rats (n = 9) under isoflurane anesthesia. Blood viscosity was analyzed by free oscillation rheometry using the instrument ReoRox®4 (Medirox, Nyköping, Sweden). A third set of animals was used for subsequent molecular analysis of identified WSS regions (n = 70 for gene expression analysis; n = 6 for protein expression studies). Following CO_2_ euthanasia, the thoracic aorta was immediately removed and quickly rinsed and perfused with either RNA later or PBS. Tissue samples for gene expression analysis were incubated overnight in RNA later and stored in fresh RNA later at −80°C pending RNA extraction. Tissue samples for morphological studies were incubated for 24 hours in 4% Zn-formaldehyde (Histolab Products, Göteborg, Sweden) and kept in 70% EtOH pending paraffin embedding.

### Acquisition of Aortic Geometry and Flow Information Using MRI

Geometrical and flow information from the aortic arch were obtained using a 1.5 T whole body MRI scanner and an eight channel wrist coil (Philips Achieva, Philips Medical Systems, Best, the Netherlands). A bolus of contrast solution (50% saline and 50% Vasovist, Bayer Schering Pharma, Berlin, Germany) (0.1 ml/100 g) was given with a power injection at 0.04 ml/s followed by 1 ml saline. The contrast bolus was tracked using a dynamic 2D gradient echo sequence with a reconstruct voxel size of 1.0×1.0×40 mm and a dynamic scan time of 328 ms. Geometrical information of the aorta was obtained using a 3D contrast-enhanced gradient-echo sequence with randomly segmented central k-space ordering (CENTRA) (TR 5.8 ms, TE 2.0 ms, and flip angle 40 degrees, field of view 70×48 mm, acquisition matrix 140×99, SENSE factor 2 in phase direction). The 3D volume data was reconstructed to a resolution of 0.3×0.3×0.3 mm. Time-resolved information of the aortic blood flow was obtained by acquisition of a 2D through-plane phase-contrast MRI sequence placed supra coronary perpendicular to the flow direction. Retrospective cardiac gating and respiratory gating were performed using an ECG trigger and respiratory belt, respectively, (SA Instruments, Inc., Stony Brook, NY). A 3 mm thick slice was acquired with a spatial resolution of 0.5×0.5 mm and a temporal resolution of approximately 18 ms (TR 9 ms, TE 3.4 ms, flip angle 15 degrees, no SENSE). Acquired data were reconstructed to 38 time frames per 2 heart cycles with a spatial resolution of 0.39×0.39 mm. The velocity data was corrected for effects of concomitant gradient fields and eddy currents.

### WSS Estimations Using Computational Fluid Dynamics

The Image material from MRI acquisitions was processed in the segmentation software Segment [14] (http://segment.heiberg.se/), using the general segmentation tool with a fast 3D level-set approach. In order to reduce the roughness of the surface due to voxel based images and level-set segmentation, i.e. gain a good 3D geometrical description of the aorta, the segmented part was smoothed using a Gaussian smoothing filter included in the segmentation software. Final geometries were trimmed in order to get appropriate surfaces for inlet (ascending aorta) and outlets (descending aorta and the three major branches in the aortic arch – innominate artery (IA), the left common carotid artery (LCCA) and the left subclavian artery (LSA)). Non-structured tetra-based meshes were created using Ansys ICEM 10.0 (ANSYS, Inc., Canonsburg, Pennsylvania, USA), with prism layers near the wall to ensure sufficient resolution for velocity gradients. This was done in order to estimate WSS with high accuracy. To ensure mesh independent results, mesh independent tests were performed.

The Computational Fluid Dynamics (CFD) simulations were performed using the commercial software Ansys FLUENT 12.0.1 (Fluent Inc., Lebanon, New Hamshire, USA). The flow was assumed to be laminar (MRI flow measurement gave a peak systole Reynolds number of approximately 350) and the wall was considered rigid with a no-slip boundary condition. Inflow for all aortas was set to a uniform velocity profile with a temporal distribution of the cardiac cycle based on the MRI measured flow of one representative rat. Roughly, the magnitude of WSS is proportional to the flow velocity, meaning that absolute magnitude of WSS would potentially differ if animal-specific flow information were to be used. However, as we are interested in the distribution of WSS, rather than the absolute magnitude, this will not affect the results. The choice of a uniform velocity profile was made based on previous findings in the human aorta, showing only a very small difference in WSS at peak systole between uniform and spatially measured velocity profile [15]. For the four outlets, temporally fixed outflow fraction boundary conditions were assigned, the fractions being 16% (IA), 7% (LCCA), 3% (LSA) and 74% (descending aorta) (based on MRI flow measurements of the complete set of rats). The blood was modeled as an incompressible Newtonian fluid with constant density and dynamic viscosity of 1050 kg/m^3^ and 0.0102 kg/ms, respectively. In order to eliminate temporal initialization effects, simulations were carried out for three cardiac cycles, using the third cardiac cycle for WSS evaluation. WSS magnitude and WSS vector direction was extracted from four time points during the cardiac cycle; late diastole, early systole, peak systole and late systole, and evaluated respectively.

### Isolation of Total RNA

With guidance from the CFD simulations, regions exposed to high and low WSS, respectively, were cut out and directly put in pre-chilled Lysing Matrix D tubes (MP Biomedicals, Illkirch, France) containing Trizol (Invitrogen, Paisley, Scotland, UK). In order to yield significant amounts of RNA, tissue pieces from the high and low WSS region, respectively, from five animals were pooled to obtain a paired sample (in total, 70 animals were used to obtain 14 pairs (i.e. 28 pools) of high and low WSS). Samples were homogenized with FastPrep. Total RNA was isolated using RNeasy Mini kit (Qiagen, Maryland, USA), including DNase treatment for elimination of potential DNA contamination. RNA integrity was analyzed by the use of an Agilent 2100 Bioanalyzer (Agilent Technologies Inc., Paolo Alto, CA, USA) and quantified using NanoDrop (NanoDrop products, Wilmington, DE, USA).

### Quantitative Real-time Polymerase Chain Reaction (QRT-PCR)

A total of 200 ng RNA from each sample was reversed transcribed with random primers and Superscript II (Invitrogen, Carlsbad, CA, USA). Amplification of cDNA was performed in 20 µl reactions using 1×TaqMan Universal PCR Mastermix (Applied Biosystems, Foster City, CA, USA) on a StepOnePlus™ Real-Time PCR System (Applied Biosystems, Foster City, CA, USA). Each sample was analyzed in duplicates and standard curve methodology was used for quantification of specific gene targets. The following Assay on Demand kits (Applied Biosystems, Foster City, CA, USA) were used: AVPR1A: Rn00583910; EMB: Rn00586815; FGF12: Rn00590748; HAND2: Rn00575515; MOBKL1A: Rn01414217; RYR3: Rn01486097; SLAIN2: Rn1765810; TNF: Rn00562055; TRPC4: Rn00584835; VCAM1: Rn00563627. TATA-binding protein (TBP, Rn01455646) served as an RNA loading control. Reactions without template were included as negative controls. The thermal protocol were as follows: 50°C for 2 min, 95°C for 10 min, followed by 50 repeats of 95°C for 15 sec, 60°C for 1 min. Expression was measured in 14 pairs of low and high WSS.

### Gene Arrays and Analysis of RNA Microarray Data

RNA samples were hybridized and scanned at the Karolinska Institute microarray core facility. Affymetrix Rat Gene 1.1 ST Array Plate system and protocols were used. Transcriptional profiling was performed on all 28 sample pools (14 pairs). Raw data from cel-files was pre-processed using the RMA-algorithm [16], in which the distribution of gene expression levels on individual arrays are normalized to the overall mRNA level distribution. Gene annotation was downloaded from the Affymetrix web page (version RaGene-1_1-st-v1.na31.rn4). Probe sets without annotation and probe sets with mean expression level below 4 were omitted from analysis, resulting in the analysis of 13 968 genes. Principal component analysis (PCA) was performed on the gene expression of all genes, using the *made4* package as implemented in R 2.13.0. Molecular function was assigned using databases of Gene Ontology by the free access DAVID algorithm http://david.abcc.ncifcrf.gov/. Multiple testing correction was inherently considered in the DAVID algorithm through the use of Benjamini-Hochsberg multiple testing correction. The microarray data is available at Gene Expression Omnibus, accession number GSE40170.

### Immunostaining

Immunostaining for HAND2, TRPC4, FGF12, Von Willebrand Factor (vWF) and PECAM1 was performed on deparaffinised tissue sections treated with DIVA solution (Biocare Medical, Concord, CA) using goat-anti-mouse HAND2 polyclonal antibody (sc-9409, Santa Cruz, UK), goat-anti-human TRPC4 polyclonal antibody (sc-15063, Santa Cruz, UK), goat-anti-human FGF12 polyclonal antibody (sc-16809, Santa Cruz, UK), rabbit-anti-human vWF polyclonal antibody (A0082, DakoCytomation, Glostrup, Denmark) or goat-anti-human PECAM1 polyclonal antibody (sc-1506, Santa Cruz, UK). PBS was included as control. Endogenous peroxidase activity was quenched with 3% hydrogen peroxide for 5 min and nonspecific binding sites were blocked with 20% goat serum. Biotinylated anti-goat or anti-rabbit IgG (Vector, Peterborough, UK) were used as secondary antibodies. Sections were then incubated with Avidin-biotin peroxidase complex (Vectastain ABC kit, Vector Laboratories, Burlingame, CA) for 30 min in room temperature, followed by visualization using 3,3′-diaminobenzidine tetrahydrochloride (Dako, Glostrup, Denmark). All sections were counterstained with Mayer’s hematoxylin (Histolab Products, Göteborg, Sweden), and blindly evaluated by three independent observers.

### Statistical Analysis

Differential expression of all 13 968 genes were investigated using a paired Student’s T-test assuming unequal variance, followed by Bonferroni correction for multiple testing. To determine if our findings were dependent of the amount of SMCs present in each sample, a linear regression analysis was performed including the expression levels of the SMC specific markers SM22 and Cnn1 as covariates, and with WSS as response variable. Statistical analyses were performed using SPSS 15.0 for Windows software (SPSS Inc., Chicago, IL, USA) or R 2.13.0.

## Results

### Geometry Data and Blood Flow in the Rat Aorta

**Figure 1:**
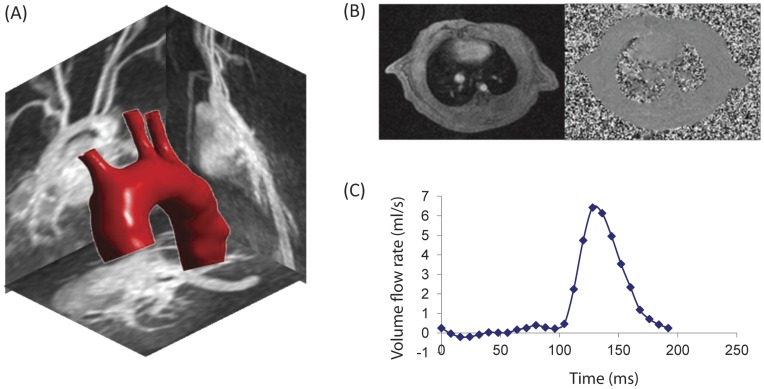
Geometrical and flow information from the rat aortic arch acquired using Magnetic Resonance Imaging. (A) A 3D surface description of the vessel wall of one representative rat aorta following image segmentation on the MRI image material, with maximum intensity projections in the sagittal, axial and coronal directions. The upper right panel (B) shows a phase-contrast and magnitude image at peak systole. The lower right panel (C) shows time-resolved ascending aortic volume flow-rate, measured by MRI and used as inflow boundary condition in the CFD simulation. MRI: magnetic resonance imaging; CFD: computational fluid dynamics.

Aortic geometry and blood flow velocities were successfully acquired in nine male Wistar rats using a 1.5 T whole body MRI scanner and an eight channel wrist coil. A 3D geometry of one representative rat aorta, with maximum intensity projections in the sagittal, axial and coronal directions is shown in Fig. 1A. Fig. 1B shows a phase-contrast and magnitude image at peak systole. Time-resolved ascending aortic volume flow rate is presented in Fig. 1C. These data constituted the basis for the CFD modeling.

### WSS Measurements

**Figure 2:**
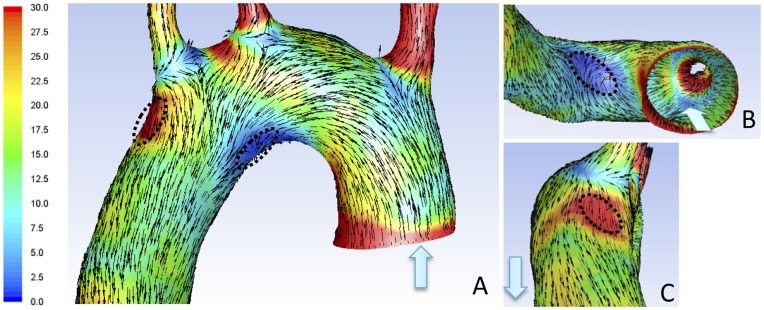
Distribution of WSS magnitude and vector direction in the rat aortic arch under physiological conditions. (A) Systolic WSS magnitude and WSS vector direction in the aortic arch (dorsal view) of one representative rat. WSS is presented as colour-coded (Pa), vector direction is presented as arrows. (B) and (C) show low (dark blue) and high (red) WSS regions, respectively, visualized from another angle. Light blue arrows indicate direction of blood flow. Dashed lines in A–C indicate regions isolated for microarray analysis. WSS: wall shear stress.

WSS magnitude and WSS direction vector were gained from the CFD simulation. The simulation was restricted to the ascending aorta, the aortic arch and a segment of the upper part of the descending aorta. In late diastole, no obvious difference in WSS magnitude was observed along the aortic arch, however, at all three systolic time points, a marked difference in WSS magnitude was seen, being consistently higher in a region along the greater curvature, just after the LSA, and lower in a region in the distal part of the lesser curvature. The difference was most prominent in peak and late systole. The WSS vector direction was at all cardiac time points uniform in high WSS region, whereas in the low WSS region, the WSS vector direction was uniform in late diastole, early systole and peak systole but divergent from the main flow direction in late systole. A 3D distribution of systolic WSS magnitude and WSS vector direction of one representative rat aorta is shown in Fig. 2. Dashed lines mark the high WSS region with a uniform flow pattern (i.e. a uniform WSS vector direction) (red area), and the low WSS region with a disturbed flow pattern (i.e. a non-uniform WSS vector direction) (dark blue area). Figure 2B and C show low and high WSS regions, respectively, visualized from another angle. Of note, WSS magnitude and WSS vector direction were analyzed and evaluated in nine Wistar rats, all showing similar general WSS pattern. The WSS magnitude ranged between 25–0 Pa in the low WSS region with disturbed flow, and between 150–60 Pa in the high WSS region with uniform flow pattern when looking at all nine animals in peak systole. Further, expanding and reducing the geometrical model of the aorta altered the absolute magnitude of WSS, becoming lower in large geometrical models and higher in small geometrical models, but had no impact on either the site of location of the two flow regions or the WSS vector direction. Hence, possible errors introduced by the MRI resolution and/or the segmentation approach will not affect the accuracy of the CFD-identified flow regions.

### Confirmation of Flow Regions

**Figure 3:**
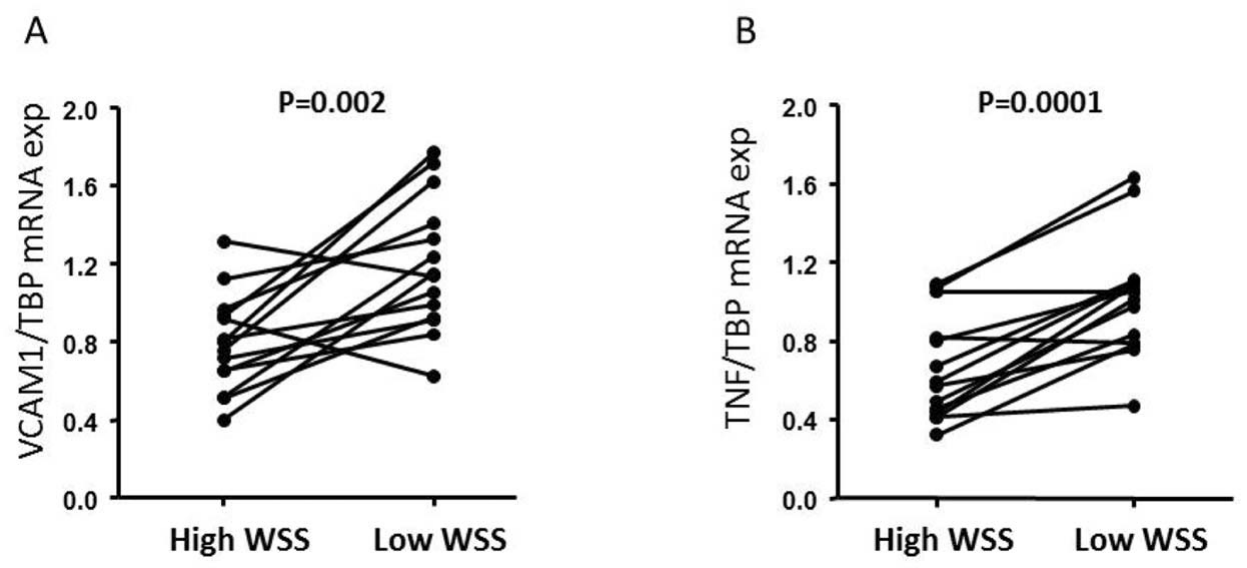
Increased expression of VACM and TNF in regions exposed to low WSS. Expression of *VCAM1* (A) and *TNF* (B) in high and low systolic WSS regions in the rat aortic arch. Gene expression was analysed by real-time PCR and normalized to *TBP* mRNA expression prior analysis (paired T-test) (n = 14 pairs). WSS: wall shear stress.

In order to confirm accurate identification and isolation of the two flow pattern regions the mRNA expression of *TNF* and *VCAM1*, two pro-inflammatory genes known to be up-regulated by low shear stress and disturbed flow [9], [17], were analyzed using QRT-PCR. Indeed, the expression of *TNF* and *VCAM1* were higher in the low WSS region than in the high WSS region (P = 0.002 for *VCAM1*; P = 0.0001 for *TNF*) (Fig. 3).

### Discovery of Mechanosensitive Genes in the Rat Aortic Arch

**Figure 4:**
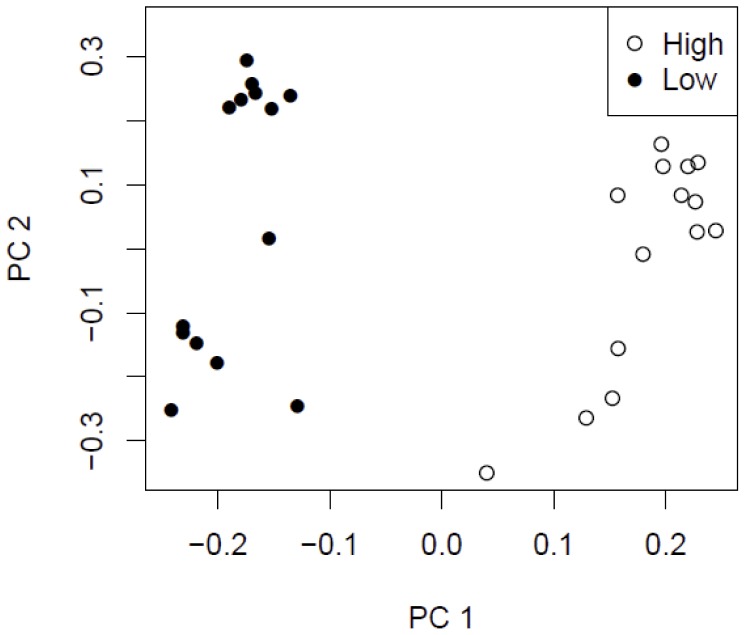
A strong differential expression between low and high WSS regions. Principal component analysis of Affymetrix Rat Gene 1.1 ST Array probe set data containing a total of 13 968 genes expressed in aortic tissue from 28 rat samples. Closed circles indicate low systolic WSS samples; open circles indicate high systolic WSS samples. The expression data was mean centred and scaled to unit variance prior analysis. WSS: wall shear stress.

To further explore the hypothesis that different flow regimes and WSS magnitudes induce distinct patterns of gene expression, mRNA from portions of the entire aortic wall exposed to high and low WSS, respectively, were subjected to global microarray analysis using the Affymetrix Rat Gene 1.1 ST Array. PCA was then applied to the Affymetrix gene array mRNA data, including 28 samples (14 paired samples of high and low WSS) and 13 968 genes. As shown in the plot (Fig. 4), we found that low (closed circles) and high (open circles) WSS samples clearly separated, indicating a strong differential gene expression between the two flow regimes. In total, 781 genes were significantly altered (P<3.6E−6; using Bonferroni correction for multiple testing), of which 387 genes (50%) were up-regulated in the low WSS region. The correlation between fold-change and log P were 0.71, thereby motivating our focus on log P values. A detailed list of all significantly altered genes is shown in Table S1. Further, the microarray analysis confirmed some of the well-known flow-sensitive genes previously reported (e.g. *klf2* and *Nfe2l2* (*Nrf2*)), and identified numerous novel mechanosensitive genes. Correction of the expression values to SMC specific markers (SM22 and calponin), in order to evaluate the potential effect of SMCs outnumbering other cell types, did not change the observed differential expression between high and low WSS samples (data not shown). In addition, we could not detect expression, or see any significant difference in expression, between high and low WSS samples of markers of infiltrating leucocytes (CD3, CD4, CD11b, CD28, CD43, CD16 and CD56) in our microarray material. Hierarchical clustering analysis of the top 50 up- and down-regulated genes demonstrated a high reproducibility of data (Figure S1).

### Functional Annotation of Mechanosensitive Genes

In order to understand the possible functional importance of the identified mechanosensitive genes, and recognize key processes involved in flow-sensitive gene expression, we performed a gene otology analysis using all the 781 significantly altered genes as input genes. The result showed that flow under physiological conditions in the rat aortic arch predominantly regulated genes involved in transcriptional regulation (P = 9.53E−08; using Benjamini Hochsberg correction for multiple testing). Specifically, *Hand2* was the most significantly altered transcription factor.

We next explored the pattern of differential expression for the top significantly altered genes, according to a cut off value of P≤1.0E−09, by performing a detailed literature search. Table 1 (including references in Table S2) shows gene symbol and level of significance for the top significantly altered genes, classified according to function. The general expression pattern was largely reminiscent of what has been reported previously, i.e. a marked up-regulation of pro-inflammatory, pro-proliferative and pro-oxidant genes, and down-regulation of anti-inflammatory, anti-proliferative and anti-oxidant related genes in low WSS regions. This further supports the accuracy of our CFD model and isolation strategy. In addition, several genes related to Ca^2+^signaling were significantly altered between the two flow regions.

**Table 1:** Top 32 mechanosensitive genes in the rat aortic arch, classified according to function(s)/properties.

**Gene symbole**	**Fold change**	**P-value**
**Calciumsignaling**		
Down-regulated*		
* Avpr1a*	2.64	1.43E−11
* Plce1*	1.50	3.73E−10
Up-regulated*		
* Trpc4*	3.38	8.72E−11
* Ryr3*	2.92	1.65E−10
* Rapgef4*	1.91	2.00E−10
**Inflammationandproliferation**		
Down-regulated*		
* Mobkl1a*	1.43	1.60E−12
* Vipr2*	2.50	1.56E−10
Up-regulated*		
* Mat2a*	1.37	3.80E−10
* Gapdh*	1.28	8.97E−10
* Pgcp*	1.74	9.65E−10
**Vascularremodeling, angiogenesis**		
Down-regulated*		
* Slain2*	3.15	1.84E−11
* Pde10a*	1.71	1.70E−10
* Figf*	3.63	2.08E−10
* Heyl*	1.69	2.69E−10
* Pax9*	2.69	3.84E−10
* Tfpi*	1.96	7.30E−10
Up-regulated*		
* Hand2*	4.06	8.91E−14
* Emb*	2.85	9.70E−12
* Capns1*	1.28	7.57E−11
**Apoptosis, endoplasmaticreticulum**		
Down-regulated*		
* Fgf12*	2.80	2.43E−12
* Rpl10a*	1.31	2.62E−11
* Gria3*	2.20	2.17E−10
* Prima1*	1.41	2.24E−10
Up-regulated*		
* Rab1b*	1.89	2.53E−11
* Ubb*	1.11	2.06E−10
* Rnf4*	1.23	3.63E−10
**Other**		
Down-regulated*		
* Atxn7l4*	1.55	7.28E−11
* Hoxb6*	2.30	1.86E−10
* Hoxa6*	2.0	8.29E−10
Up-regulated*		
* Ppib*	1.32	4.18E−11
* Stard9*	1.54	1.22E−10
* Ttc39b*	1.89	3.54E−10

* Down/up-regulated in low wall shear stress regions.

### Validation of Mechanosensitive Genes

To confirm the microarray results, QRT-PCR was performed on eight selected genes, 4 up-regulated and 4 down-regulated genes. The array data was validated for 6 of the 8 selected genes (75%); P = <0.0001 for *AVPR1A*, *EMB*, *FGF12*, *HAND2*, *RYR3* and *TRPC4*, respectively; P = 0.678 for *MOBKL1A*; P = 0.560 for *SLAIN2* (Figure S2).

**Figure 5:**
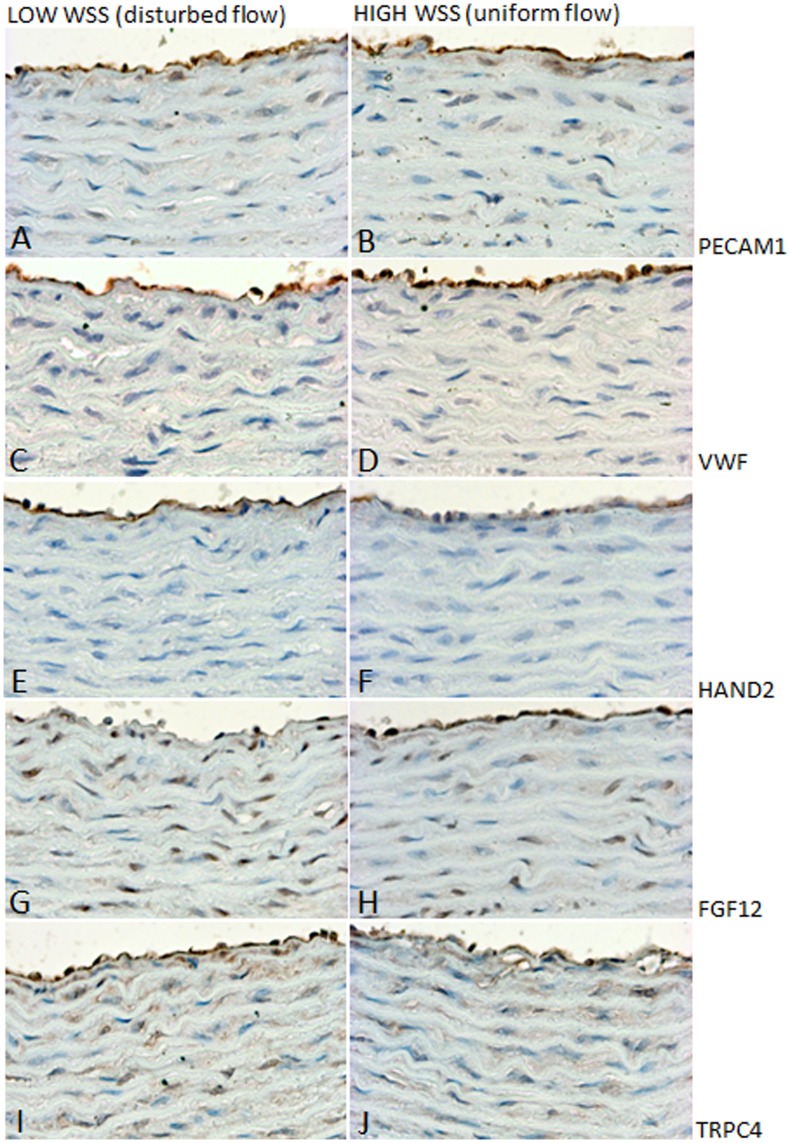
Increased expression of HAND2 and TRPC4, and decreased expression of FGF12 in regions exposed to low WSS. Immunostaining for HAND2, FGF12 and TRPC4 in low WSS/disturbed (E, G and I, respectively) and high WSS/uniform (F, H and J, respectively) flow regions, respectively. Panel A and B show staining for PECAM1, and panel C and D show staining for vWF in the corresponding regions. Sections are counterstained with Mayer’s hematoxylin. Images are representative of n = 6 animals. WSS: wall shear stress.

To further explore the validity of the flow-sensitive genes discovered in aortic regions exposed to high and low WSS, respectively, we examined protein expression of three of the genes, HAND2, FGF12 and TRPC4, using immunostaining. Paraffin embedded tissue was sectioned so that the two flow regions were located on the same section, therefore having identical staining condition. The mRNA up/down regulation of the three selected genes was confirmed for the protein expression (Fig. 5, n = 6 for each). HAND2 was specifically expressed in the endothelium, with a markedly stronger staining of HAND2 protein in low WSS regions than in the corresponding high WSS region (Fig. 5E–F). FGF12 protein expression was decreased in the endothelial layer of regions exposed to low WSS (Fig. 5G–H), as opposed to TRPC4 protein, which was higher in the low WSS regions (both in ECs and VSMCs) (Fig. 5I–J). Panel A and B show staining for PECAM1, and panel C and D show staining for vWF in the corresponding flow regions.

## Discussion

The present study investigated flow-dependent gene expression *in vivo* under physiological conditions. Using CFD, two regions being exposed to different flow patterns were identified in the rat aortic arch; the distal part of the lesser curvature being subjected to low levels of WSS and a disturbed flow pattern, and a region along the greater curvature, just after the left subclavian artery, being subjected to markedly higher levels of WSS and a uniform flow pattern. This is in line with what has been shown in mice [9], [18]. Global microarray analysis confirmed some of the previously well-known mechanosensitive genes (e.g. *klf2* and *Nrf2*) and identified numerous novel ones, particularly involved in transcriptional regulation, that to our knowledge not have been reported previously. *Hand2* was the most highly differentially expressed gene, and immunostaining revealed an endothelial specific expression of HAND2 protein, markedly higher in the regions exposed to low WSS with disturbed flow pattern.

The effect of flow on EC biology has been extensively studied *in vitro* and have contributed greatly to our understanding of EC response to fluid flow (reviewed by Chiu *et al* [2]). However, a comparison between *in vivo* and *in vitro* endothelial expression profiles demonstrated that there was only about 50% of overlap between the two [8], indicating that *in vitro* flow models may be highly simplified with non-physiological flow environments and absence of interaction between ECs and other cell layers in the vessel wall affecting the outcome. The important physiological crosstalk between ECs and underlying SMCs has particularly been shown in mice, where EC specific *KLF2* knockdown resulted in dysfunctional and disorganized SMCs [11], [12]. Importantly, a recently published report has further clarified that this effect was due to the laminar flow induced KLF2 regulation of miRNA produced in ECs, which was then transported to SMCs for modulation SMC phenotypic change [13]. Also, data from fluid modeling studies indicated that superficial SMCs may be directly exposed to magnitudes of shear stress high enough to modulate their gene expression [19], [20], regulate SMC alignment and migration [21]. These data together highlights the significant link between endothelial expression and the underlying SMC layer, and the importance of studying also concomitant events manifested in the underlying tissue in which pathological changes become most apparent.

Apart from a few studies [9], [10], [22], flow-induced gene expression in animal models have seldom been conducted under chronic and physiological conditions. On the contrary, most studies are performed in animal models with experimentally deranged shear, hence missing compensatory mechanisms operating over longer periods of time [3], [4], or in knockout animal models subjected to an atherogenic diet. Or, they lack the characterization of the regional hemodynamic environment prior determination of global gene expression [22], making it impossible to draw conclusions regarding gene expression in relation to specific flow fields. In the present work we combined CFD simulations of WSS with global microarray analysis and immunostaining of specific regions of the complete aortic wall. Certainly, the usage of full thickness vessel samples have the disadvantage of not knowing the exact cellular origin of identified flow-sensitive genes as the vessel wall contains several different cell types. In particular, identification of new endothelial-specific mechanosensitive genes may be problematic. However, what is gained by this approach is a more comprehensive picture of flow-mediated changes within the aortic wall in its natural biological context while all cell layers interact. In order to avoid alterations in gene expression due to the MRI procedure *per se,* expression analysis was performed in a second set of rats. To ensure the accuracy of CFD simulations, as well as precise isolation of the specific flow pattern regions, the expression of *TNF* and *VCAM1*, two pro-inflammatory genes known to be up-regulated by disturbed flow [9], [17], was analyzed as controls. This analysis showed that indeed, in the normal rat aortic arch, *TNF* and *VCAM1* gene expression was higher in the low WSS region compared with the high WSS region (Fig. 3), strengthening the precise identification and isolation of flow regions. Principal component analysis was further applied to the obtained microarray data and demonstrated a strong differential gene expression between the two flow pattern regions. Genes involved in transcriptional regulation were particularly regulated by fluid flow, suggesting that a change in the pattern of transcription factor activity may be a key course of action in flow-mediated changes in the aortic arch under physiological conditions. The most significantly altered gene was *Hand2.* HAND2 belongs to the bHLH family of transcription factors, and we show here that the protein expression of HAND2 was limited to the endothelial layer. Interestingly, Hath6, a protein closely related to HAND2, has also been shown to be EC specific and shear-responsive, and proposed to be important for the maintenance of flow-adaptive endothelial phenotype *in vivo* [23]. Moreover, *Hand2* has previously been reported as differentially expressed between disturbed and undisturbed flow in the normal pig aorta [22] and implicated in ECM remodeling by regulation of MMP activity [24]. MMPs function in reorganization of the ECM but can also modulate the activity of several signaling pathways by releasing growth factors or their receptors from the ECM. This has specifically been shown for MMP2, which inhibits Fgf signaling [25]. In line with this, we showed that HAND2 protein expression was markedly higher in regions exposed to low WSS relative to the corresponding high WSS regions (Fig. 5E–F), accompanied by higher expression of MMP*2* mRNA (P = 3.31E−05, not significant after Bonferroni correction) and a significantly lower expression of *Fgf12* mRNA. A decreased staining of FGF12 protein was also demonstrated in the endothelial layer of regions exposed to low WSS (Fig. 5G–H). Apart from MMP2, other MMPs (MMP11, -16, -17, -25 and -28) as well as their inhibitors, the tissue inhibitor of metalloproteinase (TIMP) 1–3 were also differentially expressed between high and low WSS regions.

Using the SABiosciences’ proprietary database (http://www.sabiosciences.com/chipqpcrsearch.php?app=TFBS), several binding sites for transcription factors (e.g. E47, HAND1, CUTL1, ATF2, HNF3β, C-JUN, FOXO1, FOXO3a and FOXO3b) were identified in a 20 kb upstream and 10 kb downstream region of the *Hand2* gene, many of which may play a role in the response of *Hand2* to shear stress. E.g., CUTL1 [26] and ATF2 have been shown to be shear-responsive and ATF2 is down-regulated by KLF2 in ECs [27]. Further, C-JUN is induced by shear stress [28] and FOXO1 has been identified as a target of KLF4 [29]. More interestingly, using a bioinformatic approach, E47, FOXO1, FOXO4 (another member of the FOXO family), as well as the HNF3β-family related transcription factor HNF4α was shown to be enriched in the upstream region of 300 genes differentially regulated by shear stress in human ECs [30]. Although these *in vitro* studies are performed in human ECs, it is reasonable to believe that these transcription factors also bind to the Hand2 promoter in rat, accounting for Hand2 regulation in response to shear stress.

In order to further explore the pattern of differential expression, a detailed literature search was performed for the top shear-sensitive genes. The general expression pattern of these were largely reminiscent of what has previously been reported for endothelial and medial cells exposed to different types of flow, i.e. up-regulation of pro-inflammatory, pro-proliferative and pro-oxidant genes, and down-regulation of anti-inflammatory, anti-proliferative and anti-oxidant related genes in regions exposed to low WSS [1], [2]. A differential expression of two of the major shear-induced transcription factors, *klf2* and *Nrf2* (also designated *Nfe2l2*) was observed (P = 0.002067 for *Klf2*; P = 9.59E−06 for *Nrf2*), although not fulfilling the stringent Bonferroni correction for multiple testing. *Klf2*, which is induced by atheroprotective flow [31], has been shown to enhance the nuclear localization of *Nrf2*, and together they explain ∼70% of the shear-sensitive gene expression variation in ECs [32], [33]. Further, a differential expression of genes related to endoplasmic reticulum (ER) stress was observed between high and low WSS regions. Shear stress has previously been shown to differentially regulate genes associated with ER stress *in vivo*, with the induction of unfolded protein response being more prominent in atherosusceptible regions [34]. Interestingly, *Prima1*, one of the genes significantly altered, has recently been robustly associated with carotid intima-media thickness in patients with atherosclerosis [35]. Moreover, the expression of *Figf (VEGFD)* was down-regulated in regions exposed to low WSS, which may seem counterintuitive as *Figf* has been reported to be angiogenic [36]. However, *Figf* has also been shown to be down-regulated in some type of cancers [37], where a pro-angiogenic state would be expected. This discrepancy may be explained by the fact that *in vivo*, several different members of the VEGF family are expressed in the cells, and that the complex process of angiogenesis is normally the outcome of a balance between all these members. In addition, Hand2 has been implicated in the regulation of angiogenesis by modulating VEGF signaling [38].

A number of genes related to Ca^2+^ signaling were significantly altered. Influx of calcium is an early response to increased shear stress, leading to elevated levels of cytosolic calcium [39], [40] with subsequent nitric oxide production. Different types of flow patterns are however expected to have different effect on the Ca^2+^ gradient [41]. TRPC4, the expression of which was up-regulated in the low WSS region both at mRNA and protein level (Fig. 5I–J), converts a variety of cellular signals into Ca^2+^ influx via activation of phospholipase C-coupled receptors [42], and has been shown to interact with the Ca^2+^ mechanosensitive channel protein polycystin 2 (PC2) [43]. Elevated expression of *TRPC4* has been suggested to contribute to vascular remodeling by modulating signaling cascades involved in cell proliferation, or by stimulating ECs to produce and release proliferative factors [44]. In addition, elevated *TRPC4* expression has been implicated in regulation of endothelial permeability by coupling the increased level of intracellular calcium to polymerization of actin and disassembly of CDH5 at gap junctions [45]. Differential expression of other genes related to Ca^2+^-signaling such as vasopressin receptor and *RYR3* also fits well into this scheme. Vasoconstrictors, such as arginine vasopressin (AVP) are known to open Ca^2+^-permeable nonselective cation channels and activate phospholipase C [46], and AVP induced Ca^2+^ oscillation is mediated through the interplay between Ca^2+^ release via RYRs and Ca^2+^ influx [47]. Taken together, in vascular areas exposed to low WSS the increased expression of *TRPC4* in the endothelium and SMCs, as well as other genes related to calcium signaling may enhance vascular remodeling and paracellular signaling, resulting in the disruption of vessel integrity and vascular dysfunction in these areas.

In the present study, a 1.5 T whole body MRI scanner was used to assess aortic geometry and time-resolved aortic blood flow information. Often, dedicated high field MRI systems are used for this purpose potentially providing better signal to noise ratio. However, in contrast-enhanced angiography, a short acquisition time at the time of contrast agent arrival is the most crucial factor. By exploiting the parallel imaging capabilities of the used receiver coil the acquisition time could be minimized. Time-resolved MRI assessment of blood velocity in rats is challenging due to limited spatial resolution and high-frequency physiological parameters. High blood velocities through small anatomies can result in displacement artifacts and signal loss in the MRI data. In the present study, signal loss was minimized by experimentally adjusting the imaging location. Displacement artifacts were limited by minimizing the echo time (TE). The obtained TE (3.5 ms) is in line with rodent studies on dedicated high field MRI systems [48]. Furthermore, using phase contrast MRI for the measurement of flow velocities, instead of e.g. ultrasound based technology, provides a high spatial resolution of velocity gradients, which in turn enables more accurate CFD simulations.

In summary, the present study investigated flow dependent gene expression in the rat aortic arch under physiological conditions. Using a CFD approach, two regions being exposed to different flow patterns were identified. Microarray analysis validated the CFD-defined flow regions and revealed a strong differential expression between high and low WSS regions, particularly associated with transcriptional regulation. Several novel mechanosensitive genes were identified, three of which were validated for protein expression. Further studies addressing the importance of specific genes in relation to atherosusceptibility are required, and may provide new insight into the understanding of flow-mediated vascular pathology.

## Supporting Information

Figure S1 Analysis of hierarchical clustering of the top 50 up- and down-regulated mechanosensitive genes shown as heat map. WSS: wall shear stress.
(TIF)

Figure S2 Expression of HAND2, FGF12, EMB, AVPR1A, RYR3, TRPC4, MOBKL1A and SLAIN2 in high and low WSS regions in the rat aortic arch. Gene expression was analysed by real-time PCR and normalized to TBP mRNA expression. ****;P<0.0001; ns: not significant. WSS: wall shear stress.
(TIF)

Table S1 Genes differentially expressed between high and low wall shear stress regions.
(XLSX)

Table S2 Top 32 mechanosensitive genes in the rat aorta, classified according to function/properties (same as Table I in manuscript, but with function(s)/properties and references).
(DOCX)

## References

[1] Nayak L, Lin Z, Jain MK (2011) “Go with the flow”: how Kruppel-like factor 2 regulates the vasoprotective effects of shear stress. Antioxid Redox Signal 15: 1449–1461. 20919941 10.1089/ars.2010.3647 PMC3144441

[2] Chiu JJ, Chien S (2011) Effects of disturbed flow on vascular endothelium: pathophysiological basis and clinical perspectives. Physiol Rev 91: 327–387. 21248169 10.1152/physrev.00047.2009 PMC3844671

[3] Wang N, Miao H, Li YS, Zhang P, Haga JH, et al (2006) Shear stress regulation of Kruppel-like factor 2 expression is flow pattern-specific. Biochem Biophys Res Commun 341: 1244–1251. 16466697 10.1016/j.bbrc.2006.01.089

[4] Willett NJ, Long RC Jr, Maiellaro-Rafferty K, Sutliff RL, Shafer R, et al (2010) An in vivo murine model of low-magnitude oscillatory wall shear stress to address the molecular mechanisms of mechanotransduction–brief report. Arterioscler Thromb Vasc Biol 30: 2099–2102. 20705917 10.1161/ATVBAHA.110.211532 PMC3148257

[5] Chien S (2008) Effects of disturbed flow on endothelial cells. Ann Biomed Eng 36: 554–562. 18172767 10.1007/s10439-007-9426-3 PMC3718045

[6] Berk BC (2008) Atheroprotective signaling mechanisms activated by steady laminar flow in endothelial cells. Circulation 117: 1082–1089. 18299513 10.1161/CIRCULATIONAHA.107.720730

[7] Garcia-Cardena G, Comander JI, Blackman BR, Anderson KR, Gimbrone MA (2001) Mechanosensitive endothelial gene expression profiles: scripts for the role of hemodynamics in atherogenesis? Ann N Y Acad Sci 947: 1–6. 11795257

[8] Ni CW, Qiu HW, Rezvan A, Kwon K, Nam D, et al (2010) Discovery of novel mechanosensitive genes in vivo using mouse carotid artery endothelium exposed to disturbed flow. Blood 116: E66–E73. 20551377 10.1182/blood-2010-04-278192 PMC2974596

[9] Suo J, Ferrara DE, Sorescu D, Guldberg RE, Taylor WR, et al (2007) Hemodynamic shear stresses in mouse aortas: implications for atherogenesis. Arterioscler Thromb Vasc Biol 27: 346–351. 17122449 10.1161/01.ATV.0000253492.45717.46

[10] LaMack JA, Himburg HA, Zhang J, Friedman MH (2009) Endothelial gene expression in regions of defined shear exposure in the porcine iliac arteries. Ann Biomed Eng 38: 2252–2262. 10.1007/s10439-010-0030-6 20387120

[11] Wu J, Bohanan CS, Neumann JC, Lingrel JB (2008) KLF2 transcription factor modulates blood vessel maturation through smooth muscle cell migration. J Biol Chem 283: 3942–3950. 18063572 10.1074/jbc.M707882200

[12] Kuo CT, Veselits ML, Barton KP, Lu MM, Clendenin C, et al (1997) The LKLF transcription factor is required for normal tunica media formation and blood vessel stabilization during murine embryogenesis. Genes Dev 11: 2996–3006. 9367982 10.1101/gad.11.22.2996 PMC316695

[13] Hergenreider E, Heydt S, Treguer K, Boettger T, Horrevoets AJ, et al (2012) Atheroprotective communication between endothelial cells and smooth muscle cells through miRNAs. Nat Cell Biol 14: 249–256. 22327366 10.1038/ncb2441

[14] Heiberg E, Sjogren J, Ugander M, Carlsson M, Engblom H, et al (2010) Design and validation of Segment–freely available software for cardiovascular image analysis. BMC Med Imaging 10: 1. 20064248 10.1186/1471-2342-10-1 PMC2822815

[15] Renner J, Loyd D, Länne T, Karlsson M (2009) Is a flat inlet profile sufficient for WSS estimation in the aortic arch? WSEAS Transactions on Fluid Mechanics 4: 148–160.

[16] Irizarry RA, Hobbs B, Collin F, Beazer-Barclay YD, Antonellis KJ, et al (2003) Exploration, normalization, and summaries of high density oligonucleotide array probe level data. Biostatistics 4: 249–264. 12925520 10.1093/biostatistics/4.2.249

[17] Mohan S, Mohan N, Valente AJ, Sprague EA (1999) Regulation of low shear flow-induced HAEC VCAM-1 expression and monocyte adhesion. Am J Physiol 276: C1100–1107. 10329958 10.1152/ajpcell.1999.276.5.C1100

[18] Janiczek RL, Blackman BR, Roy RJ, Meyer CH, Acton ST, et al (2011) Three-dimensional phase contrast angiography of the mouse aortic arch using spiral MRI. Magn Reson Med 66: 1382–1390. 21656547 10.1002/mrm.22937 PMC3170658

[19] Wang DM, Tarbell JM (1995) Modeling interstitial flow in an artery wall allows estimation of wall shear stress on smooth muscle cells. J Biomech Eng 117: 358–363. 8618390 10.1115/1.2794192

[20] Tada S, Tarbell JM (2002) Flow through internal elastic lamina affects shear stress on smooth muscle cells (3D simulations). Am J Physiol Heart Circ Physiol 282: H576–584. 11788405 10.1152/ajpheart.00751.2001

[21] Liu SQ, Tang D, Tieche C, Alkema PK (2003) Pattern formation of vascular smooth muscle cells subject to nonuniform fluid shear stress: mediation by gradient of cell density. Am J Physiol Heart Circ Physiol 285: H1072–1080. 12730056 10.1152/ajpheart.01009.2002

[22] Passerini AG, Polacek DC, Shi C, Francesco NM, Manduchi E, et al (2004) Coexisting proinflammatory and antioxidative endothelial transcription profiles in a disturbed flow region of the adult porcine aorta. Proc Natl Acad Sci U S A 101: 2482–2487. 14983035 10.1073/pnas.0305938101 PMC356976

[23] Wasserman SM, Mehraban F, Komuves LG, Yang RB, Tomlinson JE, et al (2002) Gene expression profile of human endothelial cells exposed to sustained fluid shear stress. Physiol Genomics 12: 13–23. 12419857 10.1152/physiolgenomics.00102.2002

[24] Yin C, Kikuchi K, Hochgreb T, Poss KD, Stainier DY (2010) Hand2 regulates extracellular matrix remodeling essential for gut-looping morphogenesis in zebrafish. Dev Cell 18: 973–984. 20627079 10.1016/j.devcel.2010.05.009 PMC2908152

[25] Wang Q, Uhlirova M, Bohmann D (2010) Spatial restriction of FGF signaling by a matrix metalloprotease controls branching morphogenesis. Dev Cell 18: 157–164. 20152186 10.1016/j.devcel.2009.11.004

[26] Conway DE, Williams MR, Eskin SG, McIntire LV (2010) Endothelial cell responses to atheroprone flow are driven by two separate flow components: low time-average shear stress and fluid flow reversal. Am J Physiol Heart Circ Physiol 298: H367–374. 19915176 10.1152/ajpheart.00565.2009 PMC2822569

[27] Fledderus JO, van Thienen JV, Boon RA, Dekker RJ, Rohlena J, et al (2007) Prolonged shear stress and KLF2 suppress constitutive proinflammatory transcription through inhibition of ATF2. Blood 109: 4249–4257. 17244683 10.1182/blood-2006-07-036020

[28] Nagel T, Resnick N, Dewey CF Jr, Gimbrone MA Jr (1999) Vascular endothelial cells respond to spatial gradients in fluid shear stress by enhanced activation of transcription factors. Arterioscler Thromb Vasc Biol 19: 1825–1834. 10446060 10.1161/01.atv.19.8.1825

[29] Villarreal G Jr, Zhang Y, Larman HB, Gracia-Sancho J, Koo A, et al (2010) Defining the regulation of KLF4 expression and its downstream transcriptional targets in vascular endothelial cells. Biochem Biophys Res Commun 391: 984–989. 19968965 10.1016/j.bbrc.2009.12.002 PMC4165389

[30] White SJ, Hayes EM, Lehoux S, Jeremy JY, Horrevoets AJ, et al (2011) Characterization of the differential response of endothelial cells exposed to normal and elevated laminar shear stress. J Cell Physiol 226: 2841–2848. 21302282 10.1002/jcp.22629 PMC3412226

[31] Dekker RJ, van Soest S, Fontijn RD, Salamanca S, de Groot PG, et al (2002) Prolonged fluid shear stress induces a distinct set of endothelial cell genes, most specifically lung Kruppel-like factor (KLF2). Blood 100: 1689–1698. 12176889 10.1182/blood-2002-01-0046

[32] Fledderus JO, Boon RA, Volger OL, Hurttila H, Yla-Herttuala S, et al (2008) KLF2 primes the antioxidant transcription factor Nrf2 for activation in endothelial cells. Arterioscler Thromb Vasc Biol 28: 1339–1346. 18467642 10.1161/ATVBAHA.108.165811

[33] Boon RA, Horrevoets AJ (2009) Key transcriptional regulators of the vasoprotective effects of shear stress. Hamostaseologie 29: 39–40, 41–33. 19151844

[34] Civelek M, Manduchi E, Riley RJ, Stoeckert CJ Jr, Davies PF (2009) Chronic endoplasmic reticulum stress activates unfolded protein response in arterial endothelium in regions of susceptibility to atherosclerosis. Circ Res 105: 453–461. 19661457 10.1161/CIRCRESAHA.109.203711 PMC2746924

[35] Wang L, Beecham A, Zhuo D, Dong C, Blanton SH, et al (2012) Fine mapping study reveals novel candidate genes for carotid intima-media thickness in Dominican Republican families. Circ Cardiovasc Genet 5: 234–241. 22423143 10.1161/CIRCGENETICS.111.961763 PMC3341091

[36] Marconcini L, Marchio S, Morbidelli L, Cartocci E, Albini A, et al (1999) c-fos-induced growth factor/vascular endothelial growth factor D induces angiogenesis in vivo and in vitro. Proc Natl Acad Sci U S A 96: 9671–9676. 10449752 10.1073/pnas.96.17.9671 PMC22268

[37] Metodieva SN, Nikolova DN, Cherneva RV, Dimova, II, Petrov DB, et al (2011) Expression analysis of angiogenesis-related genes in Bulgarian patients with early-stage non-small cell lung cancer. Tumori 97: 86–94. 21528670 10.1177/030089161109700116

[38] Yamagishi H, Olson EN, Srivastava D (2000) The basic helix-loop-helix transcription factor, dHAND, is required for vascular development. J Clin Invest 105: 261–270. 10675351 10.1172/JCI8856 PMC377450

[39] Jow F, Numann R (1999) Fluid flow modulates calcium entry and activates membrane currents in cultured human aortic endothelial cells. J Membr Biol 171: 127–139. 10489425 10.1007/s002329900565

[40] Helmlinger G, Berk BC, Nerem RM (1996) Pulsatile and steady flow-induced calcium oscillations in single cultured endothelial cells. J Vasc Res 33: 360–369. 8862141 10.1159/000159164

[41] Gautam M, Gojova A, Barakat AI (2006) Flow-activated ion channels in vascular endothelium. Cell Biochem Biophys 46: 277–284. 17272853 10.1385/CBB:46:3:277

[42] Rowell J, Koitabashi N, Kass DA (2010) TRP-ing up heart and vessels: canonical transient receptor potential channels and cardiovascular disease. J Cardiovasc Transl Res 3: 516–524. 20652467 10.1007/s12265-010-9208-4 PMC3875464

[43] Du J, Ding M, Sours-Brothers S, Graham S, Ma R (2008) Mediation of angiotensin II-induced Ca2+ signaling by polycystin 2 in glomerular mesangial cells. Am J Physiol Renal Physiol 294: F909–918. 18256307 10.1152/ajprenal.00606.2007

[44] Di A, Malik AB (2010) TRP channels and the control of vascular function. Curr Opin Pharmacol 10: 127–132. 20060363 10.1016/j.coph.2009.11.010

[45] Ahmmed GU, Malik AB (2005) Functional role of TRPC channels in the regulation of endothelial permeability. Pflugers Arch 451: 131–142. 15988589 10.1007/s00424-005-1461-z

[46] Pfeilschifter J, Kurtz A, Bauer C (1984) Activation of phospholipase C and prostaglandin synthesis by [arginine]vasopressin in cultures. Biochem J 223: 855–859. 6439189 10.1042/bj2230855 PMC1144372

[47] Yip KP, Sham JS (2011) Mechanisms of vasopressin-induced intracellular Ca2+ oscillations in rat inner medullary collecting duct. Am J Physiol Renal Physiol 300: F540–548. 21147839 10.1152/ajprenal.00544.2009 PMC3044002

[48] Greve JM, Les AS, Tang BT, Draney Blomme MT, Wilson NM, et al (2006) Allometric scaling of wall shear stress from mice to humans: quantification using cine phase-contrast MRI and computational fluid dynamics. Am J Physiol Heart Circ Physiol 291: H1700–1708. 16714362 10.1152/ajpheart.00274.2006

