# Supplementary figures and images for: Characterization of Shear-Sensitive Genes in the Normal Rat Aorta Identifies Hand2 as a Major Flow-Responsive Transcription Factor
Source: PLoS One. 2012 Dec 20;7(12):e52227. doi: 10.1371/journal.pone.0052227 (PMC3527404; doi:10.1371/journal.pone.0052227)

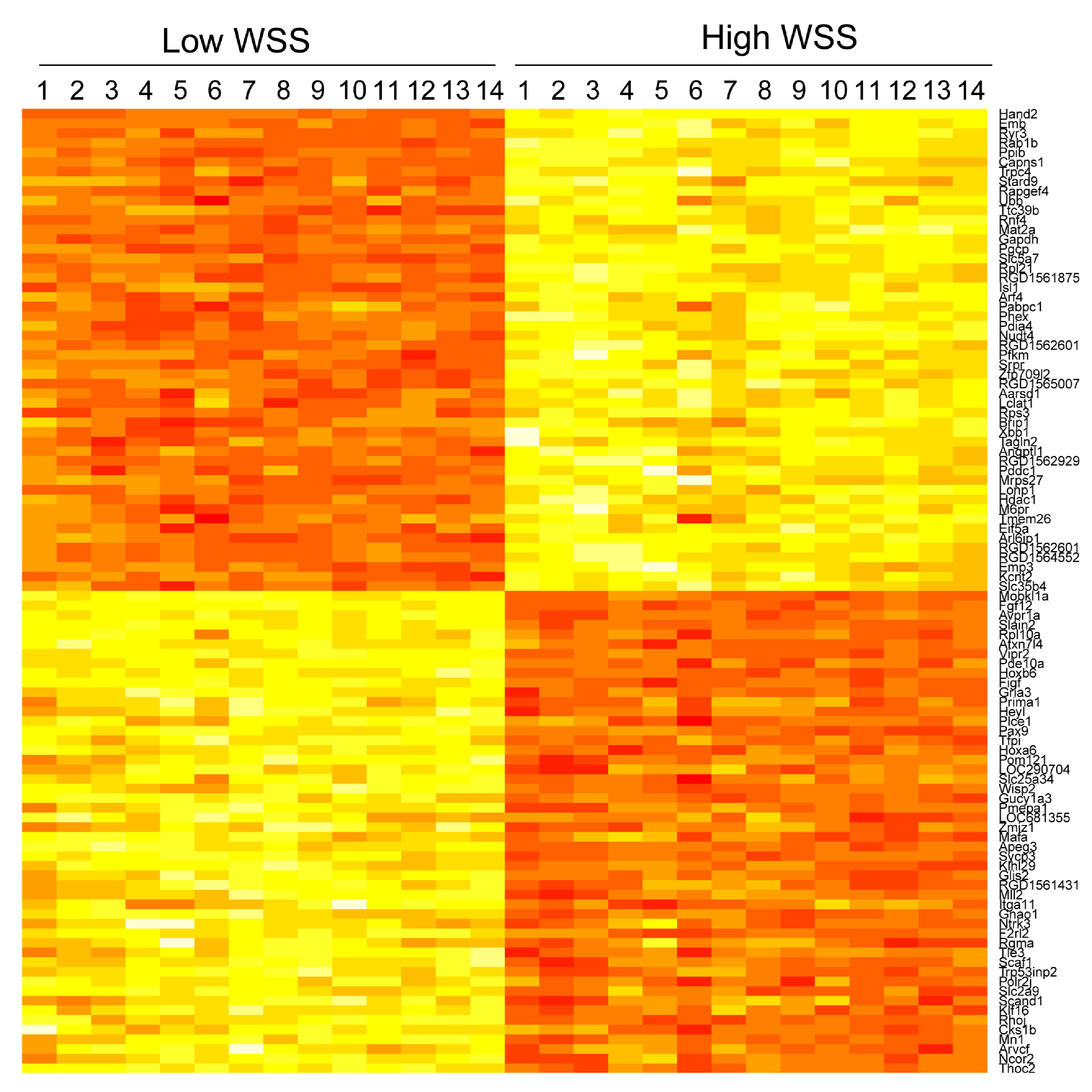

Supplement: Figure S1 — Analysis of hierarchical clustering of the top 50 up- and down-regulated mechanosensitive genes shown as heat map. WSS: wall shear stress. (TIF) [file pone.0052227.s001.tif]

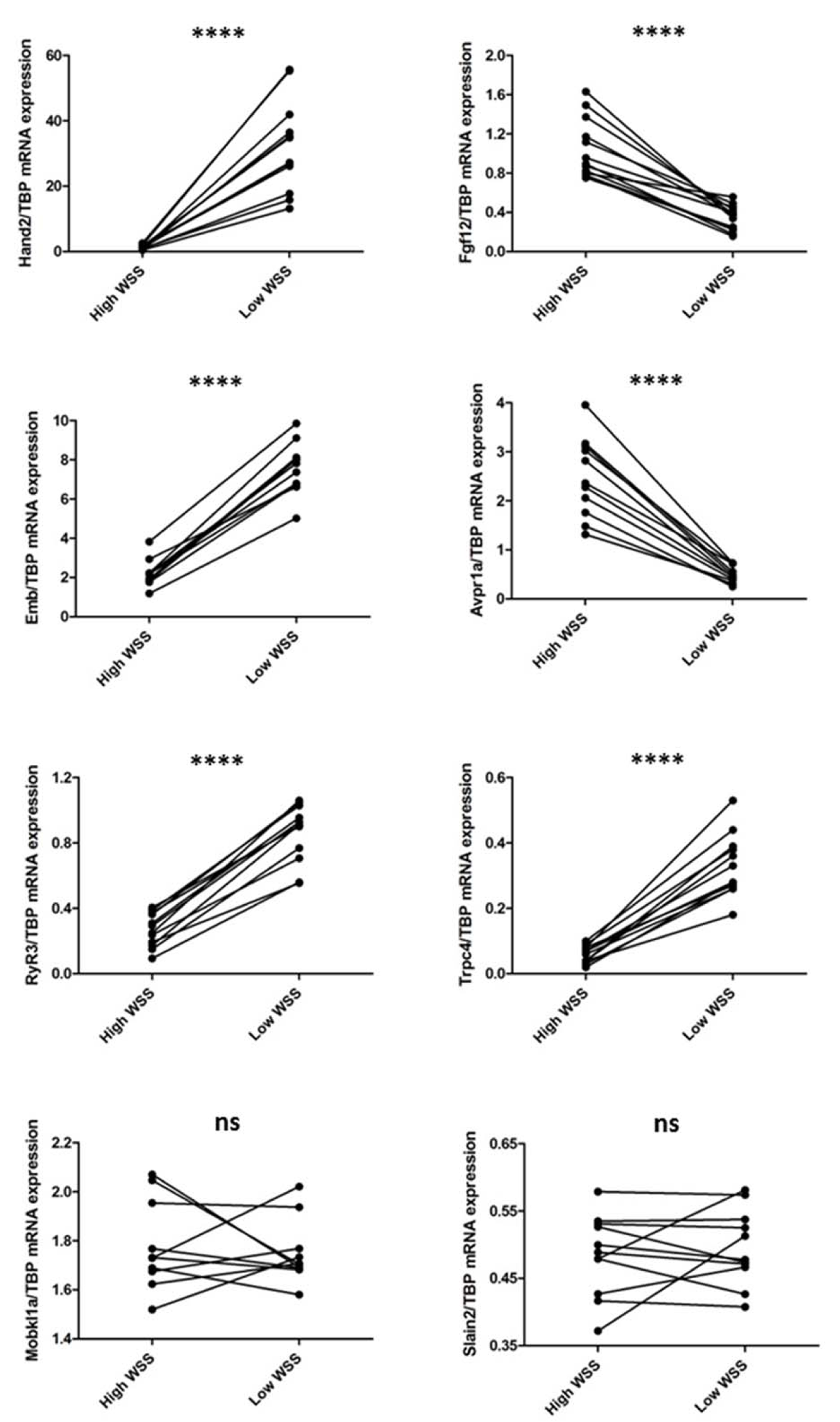

Supplement: Figure S2 — Expression of HAND2, FGF12, EMB, AVPR1A, RYR3, TRPC4, MOBKL1A and SLAIN2 in high and low WSS regions in the rat aortic arch. Gene expression was analysed by real-time PCR and normalized to TBP mRNA expression. ****;P<0.0001; ns: not significant. WSS: wall shear stress. (TIF) [file pone.0052227.s002.tif]
