# Supplementary material for: Characterization of Shear-Sensitive Genes in the Normal Rat Aorta Identifies Hand2 as a Major Flow-Responsive Transcription Factor
Source: PLoS One. 2012 Dec 20;7(12):e52227. doi: 10.1371/journal.pone.0052227 (PMC3527404; doi:10.1371/journal.pone.0052227)
Supplement: Table S2 — Top 32 mechanosensitive genes in the rat aorta, classified according to function/properties (same as Table I in manuscript, but with function(s)/properties and references). (DOCX) [file pone.0052227.s004.docx]

| **Table S2.** Top 32 mechanosensitive genes in the rat aorta, classified according to function/properties (same as Table I in manuscript, but with function(s)/properties and references). | | | | | | |
| --- | --- | --- | --- | --- | --- | --- |
| **Gene symbole** | | | **Fold change** | **P-value** | **Function(s) and properties** |  |
| **Calcium signaling** | | |  |  |  |  |
|  | Down-regulated* | |  |  |  |  |
|  |  | *Avpr1a* | 2.64 | 1.43E-11 | Vasopressin receptor, Ca^2+^ oscillation ([1](#_ENREF_1)) |  |
|  |  | *Plce1* | 1.50 | 3.73E-10 | Activation of TRPC, Ca^2+^ transport ([2](#_ENREF_2)) |  |
|  | Up-regulated* | |  |  |  |  |
|  |  | *Trpc4* | 3.38 | 8.72E-11 | Ca^2+^ transport ([3](#_ENREF_3)), PC2 interaction ([4](#_ENREF_4)) |  |
|  |  | *Ryr3* | 2.92 | 1.65E-10 | Ca^2+^ transport, PC2 interaction ([5](#_ENREF_5)) |  |
|  |  | *Rapgef4* | 1.91 | 2.00E-10 | Ca^2+^-dependent exocytosis, interacts with Plce1 ([6](#_ENREF_6)) |  |
| **Inflammation and proliferation** | | | |  |  |  |
|  | Down-regulated* | |  |  |  |  |
|  |  | *Mobkl1a* | 1.43 | 1.60E-12 | Inhibitor of proliferation ([7](#_ENREF_7)) |  |
|  |  | *Vipr2* | 2.50 | 1.56E-10 | Anti-TNF, anti-NFkB ([8](#_ENREF_8)) |  |
|  | Up-regulated* | |  |  |  |  |
|  |  | *Mat2a* | 1.37 | 3.80E-10 | Induced by TNF ([9](#_ENREF_9)) |  |
|  |  | *Gapdh* | 1.28 | 8.97E-10 | Involved in TNF-induced apoptosis ([10](#_ENREF_10)) |  |
|  |  | *Pgcp* | 1.74 | 9.65E-10 | Cell proliferation ([11](#_ENREF_11)) |  |
| **Vascular remodeling, angiogenesis** | | | | |  |  |
|  | Down-regulated* | |  |  |  |  |
|  |  | *Slain2* | 3.15 | 1.84E-11 | Microtubule growth ([12](#_ENREF_12)) |  |
|  |  | *Pde10a* | 1.71 | 1.70E-10 | Vascular remodeling ([13](#_ENREF_13)) |  |
|  |  | *Figf* | 3.63 | 2.08E-10 | VEGFD, angiogenesis ([14](#_ENREF_14)) |  |
|  |  | *Heyl* | 1.69 | 2.69E-10 | NOTCH target, cardiac valve formation ([15](#_ENREF_15)) |  |
|  |  | *Pax9* | 2.69 | 3.84E-10 | Regulated by Hand1/2 ([16](#_ENREF_16)) |  |
|  |  | *Tfpi* | 1.96 | 7.30E-10 | Anti-angiogenesis ([17](#_ENREF_17), [18](#_ENREF_18)) |  |
|  | Up-regulated* | |  |  |  |  |
|  |  | *Hand2* | 4.06 | 8.91E-14 | Cardiovascular development ([19](#_ENREF_19)), ECM remodeling ([20](#_ENREF_20)) |  |
|  |  | *Emb* | 2.85 | 9.70E-12 | Regulation of cardiac actin genes ([21](#_ENREF_21)) |  |
|  |  | *Capns1* | 1.28 | 7.57E-11 | Autophagy, angiogenesis, up-regulated by NFkB ([22](#_ENREF_22)) |  |
| **Apoptosis, endoplasmatic reticulum** | | | |  |  |  |
|  | Down-regulated* | |  |  |  |  |
|  |  | *Fgf12* | 2.80 | 2.43E-12 | Anti-apoptotic ([23](#_ENREF_23)) |  |
|  |  | *Rpl10a* | 1.31 | 2.62E-11 | UV damage and Ub/proteasome response ([24](#_ENREF_24)) |  |
|  |  | *Gria3* | 2.20 | 2.17E-10 | Anti-apoptotic ([25](#_ENREF_25)) |  |
|  |  | *Prima1* | 1.41 | 2.24E-10 | p53-dependent apoptosis ([26](#_ENREF_26)) |  |
|  | Up-regulated* | |  |  |  |  |
|  |  | *Rab1b* | 1.89 | 2.53E-11 | Small GTPase, protein transport, autophagy ([27](#_ENREF_27)) |  |
|  |  | *Ubb* | 1.11 | 2.06E-10 | Up-regulated in oxidative stress ([28](#_ENREF_28)) |  |
|  |  | *Rnf4* | 1.23 | 3.63E-10 | Ubiquitin ligase E3, regulates HIF ([29](#_ENREF_29)) |  |
| **Other** | | | |  |  |  |
|  | Down-regulated* | |  |  |  |  |
|  |  | *Atxn7l4* | 1.55 | 7.28E-11 | Unknown |  |
|  |  | *Hoxb6* | 2.30 | 1.86E-10 | Morphogenesis and development ([30](#_ENREF_30)) |  |
|  |  | *Hoxa6* | 2.0 | 8.29E-10 | Morphogenesis ([30](#_ENREF_30)), HPC development ([31](#_ENREF_31)) |  |
|  | Up-regulated* | |  |  |  |  |
|  |  | *Ppib* | 1.32 | 4.18E-11 | Pro-collagen type I chain association ([32](#_ENREF_32)) |  |
|  |  | *Stard9* | 1.54 | 1.22E-10 | Not much known |  |
|  |  | *Ttc39b* | 1.89 | 3.54E-10 | Not much known, associated with HDL-cholesterol ([33](#_ENREF_33)) |  |

* Down/up-regulated in low wall shear stress regions. ECM: Extracellular matrix; HPC: Hematopoietic progenitor cell; HIF: Hypoxia induced factor.

**References**

1. **Yip KP, Sham JS.** Mechanisms of vasopressin-induced intracellular Ca2+ oscillations in rat inner medullary collecting duct. *Am J Physiol Renal Physiol* 300: F540-548, 2011.

2. **Saleh SN, Albert AP, Large WA.** Obligatory role for phosphatidylinositol 4,5-bisphosphate in activation of native TRPC1 store-operated channels in vascular myocytes. *The Journal of physiology* 587: 531-540, 2009.

3. **Rowell J, Koitabashi N, Kass DA.** TRP-ing up heart and vessels: canonical transient receptor potential channels and cardiovascular disease. *J Cardiovasc Transl Res* 3: 516-524, 2010.

4. **Du J, Ding M, Sours-Brothers S, Graham S, Ma R.** Mediation of angiotensin II-induced Ca2+ signaling by polycystin 2 in glomerular mesangial cells. *Am J Physiol Renal Physiol* 294: F909-918, 2008.

5. **Anyatonwu GI, Ehrlich BE.** Calcium signaling and polycystin-2. *Biochem Biophys Res Commun* 322: 1364-1373, 2004.

6. **Dzhura I, Chepurny OG, Leech CA, Roe MW, Dzhura E, Xu X, Lu Y, Schwede F, Genieser HG, Smrcka AV, Holz GG.** Phospholipase C-epsilon links Epac2 activation to the potentiation of glucose-stimulated insulin secretion from mouse islets of Langerhans. *Islets* 3: 121-128, 2011.

7. **Praskova M, Xia F, Avruch J.** MOBKL1A/MOBKL1B phosphorylation by MST1 and MST2 inhibits cell proliferation. *Curr Biol* 18: 311-321, 2008.

8. **Leceta J, Gomariz RP, Martinez C, Abad C, Ganea D, Delgado M.** Receptors and transcriptional factors involved in the anti-inflammatory activity of VIP and PACAP. *Ann N Y Acad Sci* 921: 92-102, 2000.

9. **Yang H, Sadda MR, Yu V, Zeng Y, Lee TD, Ou X, Chen L, Lu SC.** Induction of human methionine adenosyltransferase 2A expression by tumor necrosis factor alpha. Role of NF-kappa B and AP-1. *The Journal of biological chemistry* 278: 50887-50896, 2003.

10. **Du ZX, Wang HQ, Zhang HY, Gao DX.** Involvement of glyceraldehyde-3-phosphate dehydrogenase in tumor necrosis factor-related apoptosis-inducing ligand-mediated death of thyroid cancer cells. *Endocrinology* 148: 4352-4361, 2007.

11. **Della Fazia MA, Piobbico D, Bartoli D, Castelli M, Brancorsini S, Viola Magni M, Servillo G.** lal-1: a differentially expressed novel gene during proliferation in liver regeneration and in hepatoma cells. *Genes Cells* 7: 1183-1190, 2002.

12. **van der Vaart B, Manatschal C, Grigoriev I, Olieric V, Gouveia SM, Bjelic S, Demmers J, Vorobjev I, Hoogenraad CC, Steinmetz MO, Akhmanova A.** SLAIN2 links microtubule plus end-tracking proteins and controls microtubule growth in interphase. *J Cell Biol* 193: 1083-1099, 2011.

13. **Tian X, Vroom C, Ghofrani HA, Weissmann N, Bieniek E, Grimminger F, Seeger W, Schermuly RT, Pullamsetti SS.** Phosphodiesterase 10A upregulation contributes to pulmonary vascular remodeling. *PLoS One* 6: e18136, 2011.

14. **Marconcini L, Marchio S, Morbidelli L, Cartocci E, Albini A, Ziche M, Bussolino F, Oliviero S.** c-fos-induced growth factor/vascular endothelial growth factor D induces angiogenesis in vivo and in vitro. *Proc Natl Acad Sci U S A* 96: 9671-9676, 1999.

15. **Luna-Zurita L, Prados B, Grego-Bessa J, Luxan G, del Monte G, Benguria A, Adams RH, Perez-Pomares JM, de la Pompa JL.** Integration of a Notch-dependent mesenchymal gene program and Bmp2-driven cell invasiveness regulates murine cardiac valve formation. *J Clin Invest* 120: 3493-3507, 2010.

16. **Barbosa AC, Funato N, Chapman S, McKee MD, Richardson JA, Olson EN, Yanagisawa H.** Hand transcription factors cooperatively regulate development of the distal midline mesenchyme. *Developmental biology* 310: 154-168, 2007.

17. **Hembrough TA, Ruiz JF, Swerdlow BM, Swartz GM, Hammers HJ, Zhang L, Plum SM, Williams MS, Strickland DK, Pribluda VS.** Identification and characterization of a very low density lipoprotein receptor-binding peptide from tissue factor pathway inhibitor that has antitumor and antiangiogenic activity. *Blood* 103: 3374-3380, 2004.

18. **Stavik B, Skretting G, Sletten M, Sandset PM, Iversen N.** Overexpression of both TFPIalpha and TFPIbeta induces apoptosis and expression of genes involved in the death receptor pathway in breast cancer cells. *Molecular carcinogenesis* 49: 951-963, 2010.

19. **Barnes RM, Firulli BA, VanDusen NJ, Morikawa Y, Conway SJ, Cserjesi P, Vincentz JW, Firulli AB.** Hand2 loss-of-function in Hand1-expressing cells reveals distinct roles in epicardial and coronary vessel development. *Circ Res* 108: 940-949, 2011.

20. **Yin C, Kikuchi K, Hochgreb T, Poss KD, Stainier DY.** Hand2 regulates extracellular matrix remodeling essential for gut-looping morphogenesis in zebrafish. *Developmental cell* 18: 973-984, 2010.

21. **Molinari S, Relaix F, Lemonnier M, Kirschbaum B, Schafer B, Buckingham M.** A novel complex regulates cardiac actin gene expression through interaction of Emb, a class VI POU domain protein, MEF2D, and the histone transacetylase p300. *Molecular and cellular biology* 24: 2944-2957, 2004.

22. **Demarchi F, Bertoli C, Copetti T, Eskelinen EL, Schneider C.** Calpain as a novel regulator of autophagosome formation. *Autophagy* 3: 235-237, 2007.

23. **Nakayama F, Muller K, Hagiwara A, Ridi R, Akashi M, Meineke V.** Involvement of intracellular expression of FGF12 in radiation-induced apoptosis in mast cells. *J Radiat Res (Tokyo)* 49: 491-501, 2008.

24. **Ferreyra ML, Biarc J, Burlingame AL, Casati P.** Arabidopsis L10 ribosomal proteins in UV-B responses. *Plant Signal Behav* 5: 1222-1225, 2010.

25. **Ripka S, Riedel J, Neesse A, Griesmann H, Buchholz M, Ellenrieder V, Moeller F, Barth P, Gress TM, Michl P.** Glutamate receptor GRIA3--target of CUX1 and mediator of tumor progression in pancreatic cancer. *Neoplasia* 12: 659-667, 2010.

26. **Lambert JM, Moshfegh A, Hainaut P, Wiman KG, Bykov VJ.** Mutant p53 reactivation by PRIMA-1MET induces multiple signaling pathways converging on apoptosis. *Oncogene* 29: 1329-1338, 2010.

27. **Zoppino FC, Militello RD, Slavin I, Alvarez C, Colombo MI.** Autophagosome formation depends on the small GTPase Rab1 and functional ER exit sites. *Traffic* 11: 1246-1261, 2010.

28. **Ryu HW, Ryu KY.** Quantification of oxidative stress in live mouse embryonic fibroblasts by monitoring the responses of polyubiquitin genes. *Biochem Biophys Res Commun* 404: 470-475, 2011.

29. **van Hagen M, Overmeer RM, Abolvardi SS, Vertegaal AC.** RNF4 and VHL regulate the proteasomal degradation of SUMO-conjugated Hypoxia-Inducible Factor-2alpha. *Nucleic Acids Res* 38: 1922-1931, 2010.

30. **Shen WF, Krishnan K, Lawrence HJ, Largman C.** The HOX homeodomain proteins block CBP histone acetyltransferase activity. *Molecular and cellular biology* 21: 7509-7522, 2001.

31. **Dickson GJ, Kwasniewska A, Mills KI, Lappin TR, Thompson A.** Hoxa6 potentiates short-term hemopoietic cell proliferation and extended self-renewal. *Exp Hematol* 37: 322-333 e323, 2009.

32. **Pyott SM, Schwarze U, Christiansen HE, Pepin MG, Leistritz DF, Dineen R, Harris C, Burton BK, Angle B, Kim K, Sussman MD, Weis M, Eyre DR, Russell DW, McCarthy KJ, Steiner RD, Byers PH.** Mutations in PPIB (cyclophilin B) delay type I procollagen chain association and result in perinatal lethal to moderate osteogenesis imperfecta phenotypes. *Hum Mol Genet* 20: 1595-1609, 2011.

33. **Kathiresan S, Willer CJ, Peloso GM, Demissie S, Musunuru K, Schadt EE, Kaplan L, Bennett D, Li Y, Tanaka T, Voight BF, Bonnycastle LL, Jackson AU, Crawford G, Surti A, Guiducci C, Burtt NP, Parish S, Clarke R, Zelenika D, Kubalanza KA, Morken MA, Scott LJ, Stringham HM, Galan P, Swift AJ, Kuusisto J, Bergman RN, Sundvall J, Laakso M, Ferrucci L, Scheet P, Sanna S, Uda M, Yang Q, Lunetta KL, Dupuis J, de Bakker PI, O'Donnell CJ, Chambers JC, Kooner JS, Hercberg S, Meneton P, Lakatta EG, Scuteri A, Schlessinger D, Tuomilehto J, Collins FS, Groop L, Altshuler D, Collins R, Lathrop GM, Melander O, Salomaa V, Peltonen L, Orho-Melander M, Ordovas JM, Boehnke M, Abecasis GR, Mohlke KL, Cupples LA.** Common variants at 30 loci contribute to polygenic dyslipidemia. *Nat Genet* 41: 56-65, 2009.
